# Improving the implementation and sustainment of evidence-based practices in community mental health organizations: a study protocol for a matched-pair cluster randomized pilot study of the Collaborative Organizational Approach to Selecting and Tailoring Implementation Strategies (COAST-IS)

**DOI:** 10.1186/s43058-020-00009-5

**Published:** 2020-02-25

**Authors:** Byron J. Powell, Amber D. Haley, Sheila V. Patel, Lisa Amaya-Jackson, Beverly Glienke, Mellicent Blythe, Rebecca Lengnick-Hall, Stacey McCrary, Rinad S. Beidas, Cara C. Lewis, Gregory A. Aarons, Kenneth B. Wells, Lisa Saldana, Mary M. McKay, Morris Weinberger

**Affiliations:** 1 Brown School, Washington University in St. Louis, One Brookings Drive, Campus Box 1196, St. Louis, MO 63130 USA; 2 Department of Health Policy and Management, Gillings School of Global Public Health, University of North Carolina at Chapel Hill, Chapel Hill, NC USA; 3 Department of Psychiatry & Behavioral Sciences, Duke University School of Medicine, Durham, NC USA; 4 National Center for Child Traumatic Stress, Durham, NC USA; 5 North Carolina Child Treatment Program, Center for Child and Family Health, Durham, NC USA; 6 School of Social Work, University of North Carolina at Chapel Hill, Chapel Hill, NC USA; 7 Department of Psychiatry, University of Pennsylvania Perelman School of Medicine, Philadelphia, PA USA; 8 Department of Medical Ethics and Health Policy, University of Pennsylvania Perelman School of Medicine, Philadelphia, PA USA; 9 Penn Implementation Science Center at the Leonard Davis Institute of Health Economics (PISCE@LDI), University of Pennsylvania, Philadelphia, PA USA; 10 MacColl Center for Health Care Innovation, Kaiser Permanente Washington Health Research Institute, Seattle, WA USA; 11 Department of Psychiatry, Child and Adolescent Services Research Center, University of California San Diego School of Medicine, San Diego, CA USA; 12 Department of Psychiatry and Behavioral Sciences, David Geffen School of Medicine, University of California Los Angeles, Los Angeles, CA USA; 13 The Jane and Terry Semel Institute for Neuroscience and Human Behavior, University of California Los Angeles, Los Angeles, CA USA; 14 Oregon Social Learning Center, Eugene, OR USA

**Keywords:** Implementation strategies, Intervention mapping, Tailored implementation strategies, Evidence-based practice, Mental health, Children and youth

## Abstract

**Background:**

Implementing and sustaining evidence-based programs with fidelity may require multiple implementation strategies tailored to address multi-level, context-specific barriers and facilitators. Ideally, selecting and tailoring implementation strategies should be guided by theory, evidence, and input from relevant stakeholders; however, methods to guide the selection and tailoring of strategies are not well-developed. There is a need for more rigorous methods for assessing and prioritizing implementation determinants (barriers and facilitators) and linking implementation strategies to determinants. The Collaborative Organizational Approach to Selecting and Tailoring Implementation Strategies (COAST-IS) is an intervention designed to increase the effectiveness of evidence-based practice implementation and sustainment. COAST-IS will enable organizational leaders and clinicians to use Intervention Mapping to select and tailor implementation strategies to address their site-specific needs. Intervention Mapping is a multi-step process that incorporates theory, evidence, and stakeholder perspectives to ensure that implementation strategies effectively address key determinants of change.

**Methods:**

COAST-IS will be piloted with community mental health organizations that are working to address the needs of children and youth who experience trauma-related emotional or behavioral difficulties by engaging in a learning collaborative to implement an evidence-based psychosocial intervention (trauma-focused cognitive behavioral therapy). Organizations will be matched and then randomized to participate in the learning collaborative only (control) or to receive additional support through COAST-IS. The primary aims of this study are to (1) assess the acceptability, appropriateness, feasibility, and perceived utility of COAST-IS; (2) evaluate the organizational stakeholders’ fidelity to the core elements of COAST-IS; and (3) demonstrate the feasibility of testing COAST-IS in a larger effectiveness trial.

**Discussion:**

COAST-IS is a systematic method that integrates theory, evidence, and stakeholder perspectives to improve the effectiveness and precision of implementation strategies. If effective, COAST-IS has the potential to improve the implementation and sustainment of a wide range of evidence-based practices in mental health and other sectors.

**Trial registration:**

This study was registered in ClinicalTrials.gov (NCT03799432) on January 10, 2019 (last updated August 5, 2019).

Contributions to the literature

- This study protocol describes an implementation intervention called the Collaborative Organizational Approach to Selecting and Tailoring Implementation Strategies (COAST-IS), which involves working with organizational leaders and clinicians to tailor implementation strategies to their site-specific needs.
- COAST-IS addresses the need for more systematic approaches for identifying and prioritizing implementation determinants and selecting implementation strategies to address them.
- COAST-IS uses Intervention Mapping, a rigorous method for developing interventions and implementation strategies, in an innovative way by engaging organizational leaders and clinicians in selecting and tailoring implementation strategies.
- COAST-IS addresses the need for systematic methods for designing and tailoring organizational-level implementation strategies.

## Background

Strengthening the public health impact of evidence-based practices (EBPs) requires effective implementation strategies, defined as “methods or techniques used to enhance the adoption, implementation, sustainment, and scale-up of a program or practice” [1, 2]. Over 70 discrete implementation strategies (e.g., audit and feedback, facilitation, supervision) have been identified [3, 4], and evidence of effectiveness for specific strategies is emerging [5–8]. However, there are no “magic bullets” [9], and the effect sizes of the most frequently used strategies are modest [5]. Increasing the effectiveness of EBP implementation might require selecting multiple discrete strategies that are tailored to address multi-level, context-specific determinants (i.e., barriers and facilitators) [10–15].

Ideally, the selection and tailoring of implementation strategies would be guided by theory, evidence, and input from relevant stakeholders [16–18]; however, the literature suggests that this is seldom the case. Implementation strategies have not often been informed by relevant theories and frameworks [8, 19–21], and poor reporting of primary research [1, 22] has made it difficult to determine the extent to which strategies are informed by evidence or involvement of appropriate stakeholders. It is also not clear whether implementation strategies used in implementation trials and applied implementation efforts address identified determinants [13, 23–26]. For example, one study of children’s mental health organizations [27] demonstrated that implementation strategies were not guided by theory or evidence, were not applied at the frequency and intensity required to implement EBPs effectively, and did not address key determinants related to the implementation process and organizational context [23, 26]. Bosch and colleagues [24] synthesized 20 studies that attempted to prospectively tailor implementation strategies to identified determinants and found that implementation strategies often were poorly conceived, with incongruence between strategies and determinants (e.g., organizational-level determinants were not addressed with organizational-level strategies). Similarly, a Cochrane systematic review concluded that while tailored implementation strategies can be effective, the effect is variable and tends to be small to moderate; it remains unclear how (1) determinants should be identified, (2) decisions should be made on which determinants are most important to address, and (3) strategies should be selected to address the important determinants [13]. This signals a need for more rigorous processes and methods to guide these key steps of implementation strategy selection and tailoring [13, 17, 18], particularly as it relates to organizational and system change efforts [18]. While several promising methods for selecting and tailoring implementation strategies have been identified [17, 18], evaluating these methods’ acceptability, appropriateness, feasibility, and the extent to which they can enhance the speed and quality at which EBPs are implemented remains a high priority [13, 17, 18, 25, 28].

The Collaborative Organizational Approach for Selecting and Tailoring Implementation Strategies (COAST-IS) is an intervention designed to increase the efficiency and effectiveness of EBP implementation and sustainment. It involves coaching organizational leaders and clinicians to use an Intervention Mapping approach [29, 30] to select and tailor implementation strategies that address their unique contextual needs. Intervention Mapping is a multi-step process that incorporates theory, evidence, and stakeholder perspectives to ensure that intervention components effectively address key determinants of change [15, 29, 30]. Intervention Mapping is an established method for developing health promotion interventions [29], but it has been underutilized in research to inform the selection and tailoring of implementation at the organizational and system levels [15, 18]. Intervention Mapping was selected to be a fundamental component of the COAST-IS intervention for three primary reasons. First, it is a promising means of strengthening the linkage between identified determinants and implementation strategies [17, 30, 31]. Second, it addresses a key priority for implementation science by explicitly identifying potential mechanisms by which implementation strategies exert their effects, shedding light on how and why they succeed or fail in achieving their intended outcomes [28, 30, 32–34]. Third, it is consistent with calls for broader stakeholder participation in the design and execution of implementation strategies [16, 35, 36], as it typically involves engaging diverse stakeholders in the Intervention Mapping process [29]. The involvement of multiple stakeholder groups will improve the rigor and relevance of this approach by including collaborations with organizations that disseminate EBPs nationally and at the state level; advisory boards comprised relevant organizational leaders and clinicians, caregivers, and youth; and organizations currently attempting to implement an EBP. Work with stakeholders will be guided by principles of community engagement, including mutual respect, two-way knowledge exchange, co-leadership/power-sharing, and trust [37–39].

This protocol paper outlines the procedures for a matched-pair cluster randomized pilot study that will (1) assess the acceptability, appropriateness, feasibility, and perceived utility of COAST-IS; (2) evaluate the organizational stakeholders’ fidelity to the core elements of COAST-IS; and (3) demonstrate the feasibility of testing COAST-IS in a larger effectiveness trial.

### Guiding conceptual models

**Fig. 1:**
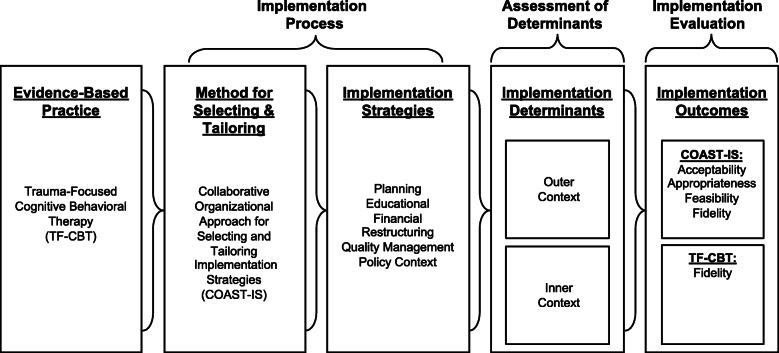
Conceptual model for the COAST-IS study. Note: the conceptual model for the COAST-IS study draws upon Proctor et al.’s [87] conceptual model for implementation research, Intervention Mapping [29], and the Exploration, Preparation, Implementation, and Sustainment model [10]

The plethora of conceptual frameworks pertinent to implementation science and practice largely serve three purposes: guide the implementation process, assess the determinants, and evaluate the implementation outcomes [40]. This study relies upon three different frameworks to accomplish those purposes. First, the COAST-IS intervention along with its core method (Intervention Mapping [29]) and the resultant implementation strategies will guide the overall process of implementation (described below). Second, the Exploration, Preparation, Implementation, and Sustainment (EPIS) model [10] will guide the assessment of determinants. The EPIS framework specifies the internal and external determinants for an organization (inner context and outer context) across four phases of the implementation process (exploration, preparation, implementation, and sustainment). During implementation, for instance, inner context factors such as organizational culture [41], organizational climate [41], and attitudes toward EBPs [42] are identified as key determinants. Outer context determinants include sociopolitical factors, funding, engagement with treatment developers, and leadership. The EPIS framework was selected because it was developed to inform implementation research in public service sectors (e.g., public mental health and child welfare services), is widely used within the field of child and adolescent mental health as well as other formal health care settings in the USA and internationally, and has identified the importance of “bridging factors” (e.g., partnerships/collaborations) that describe the relationships and activities that link outer and inner contexts [43]. Finally, the Implementation Outcome Framework [44], which specifies eight distinct outcomes, will guide the conceptualization and measurement of implementation outcomes. Implementation outcomes are useful to assess stakeholders’ perceptions of interventions and the extent to which they are implemented and sustained with quality. While they typically are assessed in relation to EBPs, they can also be applied to implementation interventions. In this study, implementation outcomes will be assessed in relation to COAST-IS (stakeholders’ perceptions of acceptability, appropriateness, feasibility, and ability to implement with fidelity) as well as assessing clinicians’ fidelity to an EBP—trauma-focused cognitive behavioral therapy (TF-CBT). The working conceptual model for this study (Fig. 1) depicts (1) the implementation of an EBP (TF-CBT [45]), (2) an innovative method for selecting and tailoring implementation strategies (COAST-IS), (3) implementation strategies that will address, (4) multi-level determinants based upon an EPIS-guided assessment [10], and (5) assessment of implementation outcomes [44] specific to COAST-IS and TF-CBT.

## Methods

### Study context, primary research partners, and the development of COAST-IS

COAST-IS will be piloted with community mental health organizations that are working to address the needs of children and youth who experience trauma-related emotional or behavioral difficulties. Children and youth experience trauma at alarming rates, which can lead to serious mental health problems including post-traumatic stress disorder, behavioral problems, depressive symptoms, and anxiety [46–49]. TF-CBT [45] is an EBP [50–52] for those who experience trauma-related emotional or behavioral difficulties. However, much like other EBPs [53–56], TF-CBT is underutilized, and even when organizations and systems adopt it, implementation problems can limit its reach and effectiveness [57–59]. The North Carolina Child Treatment Program [60], the primary research partner for this study, facilitates the implementation of trauma-focused interventions across North Carolina, largely using the National Center for Child Traumatic Stress learning collaborative model [61]. COAST-IS will be piloted within the context of two North Carolina Child Treatment Program TF-CBT learning collaboratives [62]. It is particularly appropriate to pilot COAST-IS within the context of an effort to disseminate and implement TF-CBT for two reasons: (1) it is an EBP that is a focus for wide dissemination in both specialty trauma programs and community mental health organizations across the country, and (2) it is a complex, psychosocial intervention; thus, lessons learned about using COAST-IS within this context are likely to be generalizable to other complex interventions.

Given the critical role of partnership in implementation science and practice [16, 39, 43, 63], COAST-IS was developed in partnership with the North Carolina Child Treatment Program and the US Substance Abuse and Mental Health Services Administration-funded National Center for Child Traumatic Stress. Leaders from both groups informed the development of COAST-IS through regular meetings (~monthly) and feedback on a three-part webinar series delivered by one of the authors (BJP) on implementation strategies, the need to systematically select and tailor implementation strategies, and the initial idea for the COAST-IS intervention.

Leaders from the North Carolina Child Treatment Program and the National Center for Child Traumatic Stress also helped the investigative team to form three advisory boards comprised of organizational leaders and clinicians, caregivers, and youth. The Organizational Advisory Board comprised eight organizational stakeholders similar to potential research participants. It held four 2-h meetings to review the draft intervention materials and provide feedback on the structure and content of the COAST-IS intervention. The *Family and Youth Insight Advisory Group* and *Youth Task Force* were formed to incorporate the perspectives of families and youth during intervention development. Each group comprised eight to ten caregivers or youth who had experience with trauma-focused treatment. Each group met twice for 1.5–2 h and was guided through a structured brainstorming process to identify determinants of their engagement in trauma-focused treatments and recommend strategies to address those determinants. The research team synthesized those recommendations to include in intervention materials and share with future research participants to promote client-focused implementation.

### Research design and study participants

COAST-IS will be piloted in a matched-pair cluster randomized design within two North Carolina Child Treatment Program TF-CBT learning collaboratives [62]. Additional file 3 includes a CONSORT checklist detailing reporting elements for pilot or feasibility trials. Across the two locations, the learning collaboratives have accepted 26 organizations (including community mental health organizations and child advocacy centers), eight of which will be recruited for this pilot study. The study coordinator (SM) will send an email to the senior leader who applied to the learning collaborative on their organization’s behalf to describe the purpose of the study, emphasizing that participation in the study is not a condition of the learning collaborative and explaining their organization would be randomized into a control (i.e., learning collaborative only) or intervention group receiving an adjunctive intervention (COAST-IS). If an organization agrees to participate, the primary senior leader will be asked to sign a memorandum of understanding that acknowledges their commitment to the research project, emphasizes the voluntary nature of the study, and asks for a list of additional senior leaders and clinicians who are participating in the learning collaborative and/or are actively involved in TF-CBT implementation efforts at their organization. It is anticipated a total of 10–20 senior leaders and 40–60 clinicians will participate across the 8 organizations. The investigative team will create four pairs of participating organizations matched by region and average number monthly referrals for child trauma; the organizations in each pair will be randomized to learning collaborative only or learning collaborative with COAST-IS using a random number generator. Over 12 months, each organization will receive all components of the North Carolina Child Treatment Program learning collaborative model (described below). Organizations randomized to receive the COAST-IS intervention will receive additional training and coaching to help them systematically select and tailor implementation strategies.

### Interventions

#### Control (learning collaborative only)

The North Carolina Child Treatment Program utilizes a learning collaborative model [61] that the National Center for Child Traumatic Stress adapted [64, 65] from the Institute for Healthcare Improvement’s Breakthrough Series Collaborative [66]. The collaboratives are led by experts in EBP, implementation, and quality improvement. Main components include (1) four face-to-face learning sessions (2 days each) that provide clinical training in TF-CBT, (2) post-learning session action periods structured to facilitate clinicians’ application of learned skills, (3) a secure website to facilitate faculty-to-participant and peer-to-peer learning and document the use of quality improvement methods such as “plan-do-study-act” cycles, (4) fidelity monitoring and coaching, (5) an organizational “senior leader” track supporting organizational change, (6) monthly outcomes monitoring, and (7) sustainability planning. Amaya-Jackson and colleagues [61] previously described the learning collaborative in further detail, including how specific components are linked to the implementation of science literature.

#### Intervention (learning collaborative with COAST-IS)

COAST-IS is intended to promote the implementation and sustainment of EBPs by equipping organizations to systematically select and tailor implementation strategies to address their site-specific needs. This will be accomplished by working in partnership with organizations to increase their capacity (i.e., knowledge and skill) to use Intervention Mapping [29, 30] to tailor implementation strategies to address their site-specific needs. Every effort will be made to ensure that the partnership between participating organizations and the investigative team is driven by principles of community-academic partnerships and community engagement, including mutual respect, two-way knowledge exchange, co-leadership/power-sharing, and trust [37–39, 67]. These principles will be emphasized during educational and coaching sessions, and the investigative team will regularly check with senior leaders and clinicians to ensure that these principles are realized. The process of Intervention Mapping and the modes of intervention delivery that will be used to build organizational capacity to select and tailor implementation strategies are described below.

#### Intervention Mapping

Intervention Mapping draws upon evidence, theory, stakeholder input, and a systematic process to guide intervention and implementation strategy development [15, 29]. Within this study, the investigative team will draw upon step 5 of Intervention Mapping, which focuses on the intervention implementation [29] and has recently been described in more detail as “implementation mapping” [30]. COAST-IS will employ the following four tasks to tailor implementation plans for each participating organization.

##### Task 1: Conduct a needs assessment and identify relevant implementation outcomes, performance objectives, and determinants

This task begins by conducting a needs assessment to generate consensus on the types of implementation outcomes [44] (e.g., acceptability, appropriateness, feasibility, adoption, fidelity, penetration, sustainment) stakeholders would like to improve, specify performance objectives (i.e., *who* needs to change *what* in order to achieve those implementation outcomes?), and identify determinants (i.e., what will potentially influence their ability to meet those performance objectives?) [29, 30]. This study leverages both a general and site-specific approach to the needs assessment.

The general needs assessment involved preliminary work to engage stakeholders and gave the study team insight on the types of outcomes, performance objectives, and determinants that might be relevant to implementing TF-CBT. Specifically, Organizational Advisory Board members were led through an exercise of identifying performance objectives, and the Family and Youth Insight Advisory Group and the Youth Task Force were engaged to ensure that implementation determinants from caregiver and youth perspectives were identified. All responses were recorded verbatim. Concurrently, a systematic review was conducted to identify determinants of implementing evidence-based trauma-informed interventions for children and youth [68].

The site-specific needs assessment will involve primary data collection (quantitative and qualitative) to identify organization-specific determinants. Quantitative data on implementation determinants will be assessed via Qualtrics at baseline and 12 months. The measures reflect inner setting factors of the EPIS model [10], and are psychometrically sound and pragmatic (free, brief), increasing the likelihood that organizations might use them to inform ongoing improvement efforts [69]. At the individual level, attitudes toward EBP [42] will be assessed. At the organizational level, readiness for implementing change [70], psychological safety [71], prior experiences with innovation implementation [72], organizational culture (overall) [73], organizational culture (stress) [73], organizational culture (effort) [73], learning climate [73], available resources [73], implementation climate [74], implementation leadership [75], and implementation citizenship behaviors [76] will be evaluated. Qualitative data will be derived from in-person site visits to each organization receiving the COAST-IS intervention during the first 2 months of the intervention period. The site visits will involve a structured brainstorming process [77, 78] with organizational leaders and clinicians that will yield data on relevant implementation outcomes, performance objectives, and determinants. Qualitative data provide nuanced and site-specific information about organizational needs and strengths, and is particularly important in assessing outer setting factors given the absence of quantitative measures [79].

Data from both the general and site-specific needs assessments will be summarized and shared with participating organizations, affording the opportunity to view performance objectives and determinants related to implementing and sustaining TF-CBT that are common across sites as well as those that are specific to their organizations. Matrices will be developed that link outcomes, performance objectives, and specific determinants. These linkages will identify specific targets that may need to be addressed to ensure implementation and sustainment and will be the basis for selecting implementation strategies and theoretical change methods.

##### Task 2: Identify relevant implementation strategies and theoretical change methods

Organizations will work with COAST-IS coaches (BJP, ADH, and RLH) to identify the implementation strategies that are well-suited to address implementation determinants and achieve their performance objectives. Their selection will be informed by (but not limited to) a compilation of discrete implementation strategies [3, 4, 80]. Given the importance of considering the mechanisms by which strategies might have an effect [28, 32, 33], COAST-IS coaches will encourage organizational leaders and clinicians to specify how and why they expect an implementation strategy to work. In Intervention Mapping, this is referred to as the identification of theoretical change methods [29, 30, 81]. To help in articulating the mechanisms by which the strategies are intended to operate, COAST-IS coaches will help organizational stakeholders operationalize the implementation strategies using a structured set of prompts and drawing upon taxonomies of behavior change techniques [82] and methods [81]. Organizational leaders will be encouraged to prioritize implementation strategies that are likely to impact identified determinants and performance objectives and implementation strategies that can be feasibly employed within their organization during the 12-month learning collaborative.

##### Task 3: Develop implementation plans and associated materials

Organizational leaders and clinicians on each organization’s implementation team will work with COAST-IS coaches to develop an implementation plan that includes the (1) aim and purpose of the implementation effort, (2) scope of change (e.g., what organizational units are affected), (3) individual(s) responsible for carrying out each strategy, (4) timeframe and milestones, and (5) appropriate performance/progress measures [4]. There are challenges associated with reporting implementation strategies with enough detail to promote replicability in research and practice [1, 22, 83], and there is an increasing emphasis on the importance of identifying and understanding the mechanisms through which implementation strategies exert their effects [28, 32–34]. Accordingly, each implementation plan will include detailed descriptions of each implementation strategy [1] and procedures to carefully track how they are enacted [84, 85]. This will aid in planning, executing, and reporting implementation strategies.

##### Task 4: Evaluate implementation outcomes

The fourth Intervention Mapping task is to evaluate the relevant implementation outcomes identified during task 1. For the research purposes of this study, we are assessing clinicians’ fidelity to TF-CBT; however, COAST-IS coaches will work with organizational stakeholders to identify, operationalize, and measure additional implementation outcomes that they may wish to evaluate currently or in future efforts.

##### Simple example of tasks 1–4

In task 1, organizations might identify “fidelity to TF-CBT” as a relevant outcome, “clinicians agree to receive regular fidelity monitoring and feedback” as a performance objective, and “perceptions of TF-CBT” as a potential determinant. In task 2, one or more implementation strategies and theoretical change methods that address that performance objective and determinant would be identified, for example, an opinion leader [86] who might help clinicians acknowledge the value of TF-CBT and commit to receiving regular monitoring and support, drawing upon the theoretical change method of “verbal persuasion about capability” [82]. In task 3, the opinion leader strategy would be integrated into a broader implementation plan if it was found to be feasible and likely impactful for the organization. Task 4 would involve determining whether the use of an opinion leader (likely in combination with other strategies) improved fidelity to TF-CBT. This systematic process ensures that critical determinants are addressed and closes the gap in implementation science and practice related to mismatched strategies and determinants [13, 23–25].

#### Modes of intervention delivery

The COAST-IS intervention will include the dissemination of educational materials, web-based interactive education, and web-based coaching sessions.

##### Dissemination of educational materials

COAST-IS participants will receive educational materials that provide a basic overview of implementation science and practice [87–89], describe the rationale for selecting and tailoring strategies [13, 17, 18, 25], introduce Intervention Mapping and its major steps [29, 30], and a compendium of resources to assess determinants [10, 77, 90] and identify implementation strategies [4, 31, 80].

##### Web-based interactive education

Five web-based interactive education sessions will be delivered via video conference. An attempt will be made to deliver these sessions to COAST-IS organizations simultaneously; however, scheduling difficulties might necessitate multiple sessions to ensure every organization receives each session. The didactic portion of each session will be recorded to provide a resource for organizations in the event of turnover or the need for review.

The first session will provide an overview of implementation science, the rationale for systematically selecting and tailoring implementation strategies, and the COAST-IS process. The second session will focus on task 1, involving a discussion of common performance objectives and determinants that were identified across the four COAST-IS organizations. The third session will cover task 2, offering an overview of implementation strategies that may help to address commonly identified determinants and performance objectives. The fourth session will detail the development of a matrix that matches implementation outcomes, performance objectives, and determinants to implementation strategies to inform an implementation plan and will also provide guidance for tailoring implementation strategies to address organizational needs and strengths. The fifth session will describe the development of implementation plans, provide instruction for how to track and adapt implementation strategies as needed, and suggest ways of evaluating implementation outcomes.

##### Web-based coaching

After the second education session, organizations will receive bi-weekly to monthly coaching and support from COAST-IS coaches (BJP, ADH, RLH) to build their competency related to the Intervention Mapping process and the selection and tailoring of implementation strategies. At least 12 coaching sessions will be delivered via videoconference. The amount of coaching provided will vary with the organizations’ baseline capacity to implement TF-CBT, ability to progress through the steps of Intervention Mapping, and/or requests for additional support. The first five sessions will mirror the web-based interactive educational sessions in content and will last approximately 1 h. Subsequent sessions will be scheduled at least monthly and are intended to be between 15 and 60 min depending upon agency need. Brief sessions will promote cognitive activation and feasibility. Coaching sessions will be recorded to ensure quality, promote improvement among COAST-IS coaches, and to serve as further documentation of organizations’ progression through the major tasks of step 5 of Intervention Mapping.

## Study aims and methods

### Aim 1: To assess the acceptability, appropriateness, feasibility, and perceived utility of COAST-IS

#### Participants and procedures

Senior leaders and clinicians from organizations randomized to receive the COAST-IS intervention will be contacted by email and asked to complete a brief online survey. They will also be asked to participate in a 45–60-min semi-structured interview that will be conducted by a member of the study team who has experience conducting qualitative interviews. To avoid biasing responses, interviewers will not be delivering COAST-IS educational or coaching sessions. Individuals who participate in the semi-structured interviews will be compensated $50 for their time. Interviews will be recorded, transcribed verbatim, and cleaned for analysis.

#### Measures

The online survey will include demographic questions and three four-item measures that have strong psychometric and pragmatic properties: (1) acceptability of intervention measure, (2) intervention appropriateness measure, and (3) feasibility of intervention measure [91]. Semi-structured interviews (Additional file 1) will focus on these three constructs and perceived utility of COAST-IS, as well as the extent to which principles of community engagement [37–39] were actualized and if and how they influenced stakeholders’ perceptions of COAST-IS.

#### Analysis

Quantitative data will first be assessed for missing data and distributional characteristics. Qualitative data will be imported into NVivo [92] and analyzed by two researchers using qualitative content analysis, a theory-driven approach [93, 94] that has been used in a preliminary study by the principal investigator [26, 27]. Data analysis will occur in three phases: immersion, reduction, and interpretation. The immersion phase will provide the researchers a sense of “the whole” before rearranging it into smaller segments [94]. The interviewers will develop field notes after each interview to record first impressions and analytic hunches [94] and will later review recordings and transcripts to gain a better sense of these data. Memos will record initial thoughts on themes and serve as an audit trail [94, 95]. The reduction phase will involve developing and applying a codebook to transcripts to condense data into text segments that will be aggregated into broader themes. The codebook will be refined iteratively by co-coding a sample of transcripts. The coders will independently code transcripts to increase reliability and reduce bias [93, 96], with regular meetings to discuss and resolve discrepancies. Data interpretation will involve reflecting upon the data, field notes, and memos developed during the first two phases [94]. Descriptive and interpretive summaries will include direct quotations to support descriptions and analytic assertions. Analysts will return to these data to find evidence that supports or refutes the interpretation of results. Seeking “negative cases” for which the conclusions do not hold will add credibility to the findings and ensure that the analysts are not simply seeking to confirm a certain hypothesis [94, 95]. Mixed methods analyses with equal emphasis on quantitative and qualitative methods (i.e., QUAN + QUAL) will involve merging the quantitative and qualitative data in NVivo to examine the extent to which the two types of data converge [97–99].

### Aim 2: To evaluate organizational stakeholders’ fidelity to the core elements of COAST-IS

#### Participants and procedures

The four organizations’ completing key steps of COAST-IS will be independently tracked by COAST-IS facilitators (BJP, ADH, and RL). Inter-rater reliability will be calculated, and discrepancies will be discussed until consensus is reached.

#### Measures

Informed by the Stages Implementation Completion measure [100, 101], a tool was developed to assess organizations’ fidelity to COAST-IS (see Additional file 2). The measure will be used to track the date that each COAST-IS activity in each of the four EPIS phases (exploration, preparation, implementation, and sustainment [10]) is completed.

#### Analysis

Three scores will be calculated for each phase. The “duration score” is the amount of time (in days) that a site takes to complete implementation activities in a phase and is calculated by date of entry through the date of final activity completed. The “proportion score” is the percentage of activities completed within a phase. The “phase score” marks the final phase (exploration, preparation, implementation, sustainment) that a site reaches in implementation.

### Aim 3: To demonstrate the feasibility of testing COAST-IS in an effectiveness trial

#### Participants and procedures

While aim 1 focuses on assessing the stakeholders’ perceptions of COAST-IS, aim 3 will focus on establishing the feasibility of study procedures in preparation for a larger implementation effectiveness trial [102, 103]. Organizational leaders and clinicians from all eight organizations will contribute to the investigative team’s appraisal of the study procedures such as recruitment, retention, and data collection.

#### Measures

Proportions of organizations, senior leaders, and clinicians that are willing to participate and remain in the pilot study will be documented to demonstrate the feasibility of recruitment and retention procedures.

The feasibility of procedures for assessing the implementation determinants at baseline and 12 months through an online survey via Qualtrics will also be examined via response rates for senior leaders and clinicians. The following measures will be included in a survey that will be administered at baseline and 12 months: Evidence-Based Practice Attitudes Scale [42]; Organization Readiness for Implementing Change [70]; team psychological safety [71]; perceived intensity of previous innovations, perceived failure of previous innovations, innovation-targeted helplessness, and innovation fatigue [72]; inner context measures including organizational culture (overall), organizational culture (stress), organizational culture (effort), learning climate, and available resources [73]; Jacobs et al.’s [74] measure of implementation climate; Implementation Leadership Scale [75]; and Implementation Citizenship Behavior Scale [76]. Participants will receive a $25 gift card for completing the survey.

Feasibility of collecting a key implementation outcome, fidelity to TF-CBT, will be documented using procedures established by the North Carolina Child Treatment Program. Therapist fidelity will be assessed with the TF-CBT Fidelity Metric [104]. This instrument consists of 12 4-point scales (e.g., gradual exposure, cognitive processing) that allow a trainer to rate each TF-CBT component applied by a clinician within a session. Fidelity and clinical competency in the delivery of TF-CBT components will be monitored and rated by the North Carolina Child Treatment Program Master Trainers during the clinical consultation calls. An overall fidelity score will be determined by averaging the scores from each of the 12 scales. TF-CBT Master Trainers will rate the clinician fidelity for each enrolled client for each component. Fidelity will be collected and tracked via the NC Performance and Outcomes Platform, an online platform for training, treatment, and outcomes monitoring.

#### Analysis

Appropriately, this pilot study is not powered to detect between-group differences; rather, the goal is to establish the feasibility of recruitment, randomization, retention, assessment procedures, new methods, and the implementation of a novel intervention [103, 105–108]. Thus, variables will be presented in descriptive analyses (proportions for dichotomous variables, mean and SD for continuous outcomes). We will stratify by study arm and organization where appropriate, and we will examine measures for floor/ceiling effects.

### Dissemination of study findings and refinement of the COAST-IS intervention

Study findings will be disseminated through a variety of channels. First, the main findings from the pilot study and any methodological advances (e.g., descriptions of the Intervention Mapping process applied to tailoring implementation strategies, methods for prospectively tracking implementation strategies) will be published in peer-reviewed journals and presented at relevant conferences. Second, study findings will be shared with the research participants within 3–6 months of concluding data collection via a webinar that will be open to stakeholders from each of the eight organizations. In addition, COAST-IS intervention materials (e.g., recordings of the educational sessions, educational materials) will be made available to organizations within the control group, as will summaries of their organization’s assessment of implementation determinants. Third, study partners from the North Carolina Child Treatment Program and the National Center for Child Traumatic Stress will participate in two to three videoconferences to (1) review the results from the mixed methods pilot and determine whether findings are sufficiently positive for a subsequent large-scale test of COAST-IS and, if so (2) generate potential refinements, and finalize COAST-IS for subsequent testing.

## Discussion

### Potential impact of COAST-IS

The development of rigorous and practical methods for designing and tailoring implementation strategies is a critical need for the field of implementation science [13, 17, 18, 25, 28]. COAST-IS is a novel implementation intervention that responds to this need and is intended to strengthen organizations’ capacity to implement and sustain EBPs by improving the precision and effectiveness of implementation strategies. It leverages an established method for developing interventions, Intervention Mapping [29, 30], which systematically links performance objectives, determinants, and implementation strategies in a manner that is likely to improve our ability to understand, assess, and change mechanisms of effective implementation [28, 32–34]. This study will determine whether COAST-IS is an acceptable, appropriate, and feasible approach to tailoring implementation strategies at the organizational level and, if a larger-scale trial is warranted, ways in which it may need to be refined prior to further testing.

While COAST-IS is being applied to improve the implementation of TF-CBT in community mental health settings, it is intended to be broadly applicable to organizations implementing a wide range of interventions. If stakeholders are able to apply COAST-IS with fidelity, it could be used to make implementation strategies such as learning collaboratives and facilitation more systematic and transparent by clearly defining specific steps for designing and tailoring implementation strategies.

This study will also demonstrate how diverse stakeholder groups can inform the implementation of EBPs [16, 36, 39]. In this case, organizations that disseminate EBPs at the national (National Center for Child Traumatic Stress) and state (North Carolina Child Treatment Program) levels are invaluable partners that informed the conceptualization and design of COAST-IS, enabled access to organizations implementing TF-CBT, and provided clinical and implementation expertise specific to trauma-focused interventions. Organizational leaders and clinicians from the Organizational Advisory Board provided early feedback on COAST-IS and enhance the likelihood that it would be acceptable, appropriate, and feasible within the context of community mental health. Caregiver- and youth-focused advisory boards provided insight into the potential implementation determinants. Finally, the organizational leaders and clinicians that will receive COAST-IS will collaboratively select and tailor implementation strategies and have numerous opportunities to provide feedback related to its structure and content that will guide future refinements. Engaging stakeholders with a spirit of mutual respect, two-way knowledge exchange, co-leadership/power-sharing, and trust [37–39] is anticipated to increase stakeholder buy-in, improve the design of COAST-IS, and ensure that the tailored strategies are highly aligned with the needs and values of participating organizations.

### Innovation

Several innovative features of COAST-IS are worth noting. First, the use of Intervention Mapping [29, 30] to select and tailor implementation strategies in community settings and its application to mental health are innovative [15]. Also innovative is engaging organizational stakeholders to identify site-specific determinants and strategies, rather than the traditional strategy of having a central team. Second, most systematic methods to design implementation strategies have focused on individual provider behavior change; this study focuses on organizational-level change [18]. Finally, trials of tailored implementation strategies often use passive comparators (e.g., dissemination of guidelines or educational materials) [13], whereas this study sets the stage for a larger trial that would compare COAST-IS to a learning collaborative, a real-world approach adopted by an increasing number of organizations.

### Limitations

By design, this study is not able to detect between-group differences; however, this is appropriate given the primary purpose of this study is to demonstrate the feasibility of the intervention and study methods in preparation for a larger trial [103, 105–108]. Another potential limitation is that randomizing organizations within the context of two learning collaboratives makes contamination a potential threat. However, studies of TF-CBT collaboratives show that advice seeking between organizations [109] and communication patterns within organizations change minimally [110]. This threat will be further minimized by (1) asking participants not to discuss COAST-IS during cross-organizational communication during the collaborative and (2) examining meeting notes to ensure that COAST-IS is not discussed during cross-organizational learning sessions.

## Conclusion

This research addresses important national priorities outlined by the National Academies of Sciences, Engineering, and Medicine to advance the implementation of evidence-based psychosocial interventions for children, youth, and families [111, 112], as well as the National Institute of Mental Health’s Strategic Plan to increase the public health impact of their funded research [113]. It is highly responsive to the National Institutes of Health’s priorities for implementation science given its focus on developing and testing implementation strategies; understanding context and local capacity; influencing organizational climate and processes; leveraging relevant implementation frameworks; understanding potential mechanisms of change within multi-level, multi-component implementation strategies; and incorporation of a mixed methods evaluation [34]. Ultimately, it has the potential to positively impact public health by improving the implementation and sustainment of EBPs in community mental health settings by equipping organizations to systematically address context- and intervention-specific determinants of implementation and sustainment. COAST-IS addresses challenges that are common to all implementation efforts; thus, it is anticipated that lessons learned from this pilot and subsequent refinements will be relevant well beyond the field of mental health.

## Supplementary information

**Additional file 1.** Semi-Structured Interview Guide for Aim 1 of COAST-IS Study

**Additional file 2.** COAST-IS Fidelity Tool (Date of Version: 9-19-19)

**Additional file 3.** CONSORT 2010 checklist of information to include when reporting a pilot or feasibility trial*

## Data Availability

Not applicable.

## Abbreviations

COAST-IS: Collaborative Organizational Approach to Selecting and Tailoring Implementation Strategies
EBP: Evidence-based practice
EPIS: Exploration, Preparation, Implementation, and Sustainment framework
TF-CBT: Trauma-Focused Cognitive Behavioral Therapy

## References

[1] Proctor EK, Powell BJ, McMillen JC (2013). Implementation strategies: recommendations for specifying and reporting. Implement Sci.

[2] Powell BJ, Garcia K, Fernandez ME, Chambers D, Vinson C, Norton W (2019). Implementation strategies. Optimizing the cancer control continuum: advancing implementation research.

[3] Powell BJ, McMillen JC, Proctor EK, Carpenter CR, Griffey RT, Bunger AC (2012). A compilation of strategies for implementing clinical innovations in health and mental health. Med Care Res Rev.

[4] Powell BJ, Waltz TJ, Chinman MJ, Damschroder LJ, Smith JL, Matthieu MM (2015). A refined compilation of implementation strategies: results from the Expert Recommendations for Implementing Change (ERIC) project. Implement Sci.

[5] Grimshaw JM, Eccles MP, Lavis JN, Hill SJ, Squires JE (2012). Knowledge translation of research findings. Implement Sci.

[6] Landsverk J, Brown CH, Rolls Reutz J, Palinkas LA, Horwitz SM (2011). Design elements in implementation research: a structured review of child welfare and child mental health studies. Adm Policy Ment Health Ment Health Serv Res.

[7] Novins DK, Green AE, Legha RK, Aarons GA (2013). Dissemination and implementation of evidence-based practices for child and adolescent mental health: a systematic review. J Am Acad Child Adolesc Psychiatry.

[8] Powell BJ, Proctor EK, Glass JE (2014). A systematic review of strategies for implementing empirically supported mental health interventions. Res Soc Work Pract.

[9] Oxman AD, Thomson MA, Davis DA, Haynes B (1995). No magic bullets: a systematic review of 102 trials of interventions to improve professional practice. Can Med Assoc J.

[10] Aarons GA, Hurlburt M, Horwitz SM (2011). Advancing a conceptual model of evidence-based practice implementation in public service sectors. Adm Policy Ment Health Ment Health Serv Res.

[11] Mittman BS, Brownson RC, Colditz GA, Proctor EK (2012). Implementation science in health care. Dissemination and implementation research in health: translating science to practice.

[12] Weiner BJ, Lewis MA, Clauser SB, Stitzenberg KB (2012). In search of synergy: strategies for combining interventions at multiple levels. JNCI Monographs.

[13] Baker R, Comosso-Stefinovic J, Gillies C, Shaw EJ, Cheater F, Flottorp S (2015). Tailored interventions to address determinants of practice. Cochrane Database Syst Rev.

[14] Wensing M, Oxman A, Baker R, Godycki-Cwirko M, Flottorp S, Szecsenyi J (2011). Tailored implementation for chronic diseases (TICD): a project protocol. Implement Sci.

[15] Powell Byron J., Beidas Rinad S., Lewis Cara C., Aarons Gregory A., McMillen J. Curtis, Proctor Enola K., Mandell David S. (2015). Methods to Improve the Selection and Tailoring of Implementation Strategies. The Journal of Behavioral Health Services & Research.

[16] Chambers DA, Azrin ST (2013). Partnership: a fundamental component of dissemination and implementation research. Psychiatr Serv.

[17] Powell BJ, Beidas RS, Lewis CC, Aarons GA, McMillen JC, Proctor EK (2017). Methods to improve the selection and tailoring of implementation strategies. J Behav Health Serv Res.

[18] Colquhoun HL, Squires JE, Kolehmainen N, Grimshaw JM (2017). Methods for designing interventions to change healthcare professionals’ behaviour: a systematic review. Implement Sci.

[19] Colquhoun HL, Brehaut JC, Sales A, Ivers N, Grimshaw J, Michie S (2013). A systematic review of the use of theory in randomized controlled trials of audit and feedback. Implement Sci.

[20] Davies P, Walker AE, Grimshaw JM (2010). A systematic review of the use of theory in the design of guideline dissemination and implementation strategies and interpretation of the results of rigorous evaluations. Implement Sci.

[21] Williams NJ, Beidas RS (2019). The state of implementation science in child psychology and psychiatry: a review and suggestions to advance the field. J Child Psychol Psychiatry.

[22] Michie S, Fixsen DL, Grimshaw JM, Eccles MP (2009). Specifying and reporting complex behaviour change interventions: the need for a scientific method. Implement Sci.

[23] Powell BJ. A mixed methods multiple case study of implementation as usual in children’s social service organizations: study protocol: Washington University in St. Louis; 2014. Available from: http://openscholarship.wustl.edu/cgi/viewcontent.cgi?article=2335&context=etd. Accessed 6 Feb 2020. 10.1186/1748-5908-8-92 PMC3751866 23961701

[24] Bosch M, van der Weijden T, Wensing M, Grol R (2007). Tailoring quality improvement interventions to identified barriers: a multiple case analysis. J Eval Clin Pract.

[25] Wensing M (2017). The Tailored Implementation in Chronic Diseases (TICD) project: introduction and main findings. Implement Sci.

[26] Powell BJ, Proctor EK (2016). Learning from implementation as usual in children’s mental health. Implement Sci.

[27] Powell BJ, Proctor EK, Glisson CA, Kohl PL, Raghavan R, Brownson RC (2013). A mixed methods multiple case study of implementation as usual in children’s social service organizations: study protocol. Implement Sci.

[28] Powell BJ, Fernandez ME, Williams NJ, Aarons GA, Beidas RS, Lewis CC (2019). Enhancing the impact of implementation strategies in healthcare: a research agenda. Front Public Health.

[29] Bartholomew Eldridge LK, Markham CM, Ruiter RAC, Fernández ME, Kok G, Parcel GS (2016). Planning health promotion programs: an intervention mapping approach.

[30] Fernandez ME, ten Hoor GA, van Lieshout S, Rodriguez SA, Beidas RS, Parcel G (2019). Implementation mapping: using intervention mapping to develop implementation strategies. Front Public Health.

[31] Waltz TJ, Powell BJ, Fernández ME, Abadie B, Damschroder LJ (2019). Choosing implementation strategies to address contextual barriers: diversity in recommendations and future directions. Implement Sci.

[32] Lewis CC, Klasnja P, Powell BJ, Lyon AR, Tuzzio L, Jones S (2018). From classification to causality: advancing understanding of mechanisms of change in implementation science. Front Public Health.

[33] Williams NJ (2016). Multilevel mechanisms of implementation strategies in mental health: integrating theory, research, and practice. Adm Policy Ment Health Ment Health Serv Res.

[34] National Institutes of Health (2019). Dissemination and implementation research in health (R01 clinical trial optional).

[35] Metz A, Boaz A, Powell BJ (2019). A research protocol for studying participatory processes in the use of evidence in child welfare systems. Evid Policy.

[36] Palinkas LA, Short C, Wong M (2015). Research-practice-policy partnerships for implementation of evidence-based practices in child welfare and child mental health.

[37] Jones L, Wells K (2007). Strategies for academic and clinician engagement in community-participatory partnered research. JAMA.

[38] Jones L, Wells K, Norris K, Meade B, Koegel P (2009). The vision, valley, and victory of community engagement. Ethn Dis.

[39] Shea CM, Young TL, Powell BJ, Rohweder C, Enga ZK, Scott JE (2017). Researcher readiness for participating in community-engaged dissemination and implementation research. Transl Behav Med.

[40] Nilsen P (2015). Making sense of implementation theories, models and frameworks. Implement Sci.

[41] Glisson C, Landsverk J, Schoenwald S, Kelleher K, Hoagwood KE, Mayberg S (2008). Assessing the organizational social context (OSC) of mental health services: implications for research and practice. Adm Policy Ment Health Ment Health Serv Res.

[42] Aarons GA (2004). Mental health provider attitudes toward adoption of evidence-based practice: the Evidence-Based Practice Attitude Scale (EBPAS). Ment Health Serv Res.

[43] Moullin JC, Dickson KS, Stadnick NA, Rabin B, Aarons GA (2019). Systematic review of the exploration, preparation, implementation, sustainment (EPIS) framework. Implement Sci.

[44] Proctor EK, Silmere H, Raghavan R, Hovmand P, Aarons GA, Bunger A (2011). Outcomes for implementation research: conceptual distinctions, measurement challenges, and research agenda. Adm Policy Ment Health Ment Health Serv Res.

[45] Cohen JA, Mannarino AP, Deblinger E (2017). Treating trauma and traumatic grief in children and adolescents.

[46] Copeland WE, Keeler G, Angold A, Costello EJ (2007). Traumatic events and posttraumatic stress in childhood. Arch Gen Psychiatry.

[47] McLaughlin KA, Koenen KC, Hill E, Petukhova M, Sampson NA, Zaslavsky A (2013). Trauma exposure and postraumatic stress disorder in a US national sample of adolescents. J Am Acad Child Adolesc Psychiatry.

[48] Finkelhor D, Turner H, Ormrod R, Hamby SL (2009). Violence, abuse, and crime exposure in a national sample of children and youth. Pediatrics.

[49] Hillis S, Mercy J, Amobi A, Kress H (2016). Global prevalence of past-year violence against children: a systematic review and minimum evidence. Pediatrics.

[50] Silverman WK, Ortiz CD, Viswesvaran C, Burns BJ, Kolko DJ, Putnam FW (2008). Evidence-based psychosocial treatments for children and adolescents exposed to traumatic events. J Clin Child Adolesc Psychol.

[51] Cary CE, McMillen JC (2012). The data behind the dissemination: a systematic review of trauma-focused cognitive behavioral therapy for use with children and youth. Child Youth Serv Rev.

[52] Dorsey Shannon, McLaughlin Katie A., Kerns Suzanne E. U., Harrison Julie P., Lambert Hilary K., Briggs Ernestine C., Revillion Cox Julia, Amaya-Jackson Lisa (2016). Evidence Base Update for Psychosocial Treatments for Children and Adolescents Exposed to Traumatic Events. Journal of Clinical Child & Adolescent Psychology.

[53] Garland AF, Brookman-Frazee L, Hurlburt MS, Accurso EC, Zoffness RJ, Haine-Schlagel R (2010). Mental health care for children with disruptive behavior problems: a view inside therapists’ offices. Psychiatr Serv.

[54] Zima BT, Hurlburt MS, Knapp P, Ladd H, Tang L, Duan N (2005). Quality of publicly-funded outpatient specialty mental health care for common childhood psychiatric disorders in California. J Am Acad Child Adolesc Psychiatry.

[55] Kohl PL, Schurer J, Bellamy JL (2009). The state of parent training: program offerings and empirical support. Fam Soc.

[56] Raghavan R, Inoue M, Ettner SL, Hamilton BH (2010). A preliminary analysis of the receipt of mental health services consistent with national standards among children in the child welfare system. Am J Public Health.

[57] Allen B, Johnson JC (2012). Utilization and implementation of trauma-focused cognitive-behavioral therapy for the treatment of maltreated children. Child Maltreatment.

[58] Powell BJ, Hausmann-Stabile C, McMillen JC (2013). Mental health clinicians’ experiences of implementing evidence-based treatments. J Evid Based Soc Work.

[59] Rudd BN, Last BS, Gregor C, Jackson K, Berkowitz S, Zinny A (2019). Benchmarking treatment effectiveness of community-delivered trauma-focused cognitive behavioral therapy. Am J Community Psychol.

[60] NC Child Treatment Program [Internet]. 2019. Available from: https://www.ncchildtreatmentprogram.org/about-us/. Accessed 6 Feb 2020.

[61] Amaya-Jackson L, Hagele D, Sideris J, Potter D, Briggs EC, Keen L (2018). Pilot to policy: statewide dissemination and implementation of evidence-based treatment for traumatized youth. BMC Health Serv Res.

[62] Markiewicz J, Ebert L, Ling D, Amaya-Jackson L, Kisiel C (2006). Learning collaborative toolkit.

[63] Aarons GA, Fettes DL, Hurlburt MS, Palinkas LA, Gunderson L, Willging CE (2014). Collaboration, negotiation, and coalescence for interagency-collaborative teams to scale-up evidence-based practice. J Clin Child Adolesc Psychol.

[64] Ebert L, Amaya-Jackson L, Markiewicz JM, Kisiel C, Fairbank JA (2012). Use of the breakthrough series collaborative to support broad and sustained use of evidence-based trauma treatment for children in community practice settings. Adm Policy Ment Health Ment Health Serv Res.

[65] Ebert L, Amaya-Jackson L, Markiewicz J, Fairbank JA, McHugh RK, Barlow DH (2012). Development and application of the NCCTS learning collaborative model for the implementation of evidence-based child trauma treatment. Dissemination and implementation of evidence-based psychological interventions.

[66] Institute for Healthcare Improvement (2003). The Breakthrough Series: IHI’s colaborative model for achieving breakthrough improvement.

[67] Drahota A, Meza RD, Brikho B, Naaf M, Estabillo JA, Gomez ED (2016). Community-academic partnerships: a systematic review of the state of the literature and recommendations for future research. Milbank Q.

[68] Powell BJ, Patel SV, Haley AD, Haines ER, Knocke KE, Chandler S, et al. Determinants of implementing evidence-based trauma-focused clinical interventions for children and youth: A systematic review. Adm Policy Ment Health and Ment Health Serv Res. 2019. 10.1007/s10488-019-01003-3. 10.1007/s10488-019-01003-3 PMC7275881 31813066

[69] Powell BJ, Stanick CF, Halko HM, Dorsey CN, Weiner BJ, Barwick M, et al. Toward criteria for pragmatic measurement in implementation research and practice: A stakeholder- driven approach using concept mapping. 2017;12(118):1–7. 10.1186/s13012-017-0649-x PMC5627503 28974248

[70] Shea CM, Jacobs SR, Esserman DA, Bruce K, Weiner BJ (2014). Organizational readiness for implementing change: a psychosocial assessment of a new measure. Implement Sci.

[71] Edmondson AC (1999). Psychological safety and learning behavior in work teams. Adm Sci Q.

[72] Chung GH, Choi JN, Du AJ (2017). Tired of innovations? Learned helplessness and fatigue in the context of continuous streams of innovation implementation. J Organ Behav.

[73] Fernandez ME, Walker TJ, Weiner BJ, Calo WA, Liang S, Risendal B (2018). Developing measures to assess constructs from the inner setting domain of the consolidated framework for implementation research. Implement Sci.

[74] Jacobs SR, Weiner BJ, Bunger AC (2014). Context matters: measuring implementation climate among individuals and groups. Implement Sci.

[75] Aarons GA, Ehrhart MG, Farahnak LR (2014). The implementation leadership scale (ILS): development of a brief measure of unit level implementation leadership. Implement Sci.

[76] Ehrhart MG, Aarons GA, Farahnak LR (2015). Going above and beyond for implementation: the development and validity testing of the Implementation Citizenship Behavior Scale (ICBS). Implement Sci.

[77] Flottorp SA, Oxman AD, Krause J, Musila NR, Wensing M, Godycki-Cwirko M (2013). A checklist for identifying determinants of practice: a systematic review and synthesis of frameworks and taxonomies of factors that prevent or enable improvements in healthcare professional practice. Implement Sci.

[78] Krause J, Van Lieshout J, Klomp R, Huntink E, Aakhus E, Flottorp S, et al. Identifying determinants of care for tailoring implementation in chronic diseases: an evaluation of different methods. Implement Sci. 2014;9:102. 10.1186/s13012-014-0102-3 PMC4243773 25112492

[79] Lewis CC, Scott K, Marti CN, Marriott BR, Kroenke K, Putz JW (2015). Implementing measurement-based care (iMBC) for depression in community mental health: a dynamic cluster randomized trial study protocol. Implement Sci.

[80] Waltz TJ, Powell BJ, Matthieu MM, Damschroder LJ, Chinman MJ, Smith JL (2015). Use of concept mapping to characterize relationships among implementation strategies and assess their feasibility and importance: results from the Expert Recommendations for Implementing Change (ERIC) study. Implement Sci.

[81] Kok Gerjo, Gottlieb Nell H., Peters Gjalt-Jorn Y., Mullen Patricia Dolan, Parcel Guy S., Ruiter Robert A.C., Fernández María E., Markham Christine, Bartholomew L. Kay (2015). A taxonomy of behaviour change methods: an Intervention Mapping approach. Health Psychology Review.

[82] Michie S, Richardson M, Johnston M, Abraham C, Francis J, Hardeman W (2013). The behavior change technique taxonomy (v1) of 93 hierarchically clustered techniques: building an international consensus for the reporting of behavior change interventions. Ann Behav Med.

[83] Albrecht L, Archibald M, Arseneau D, Scott SD (2013). Development of a checklist to assess the quality of reporting of knowledge translation interventions using the Workgroup for Intervention Development and Evaluation Research (WIDER) recommendations. Implement Sci.

[84] Bunger AC, Powell BJ, Robertson HA, MacDowell H, Birken SA, Shea C (2017). Tracking implementation strategies: a description of a practical approach and early findings. Health Res Policy Syst.

[85] Boyd MR, Powell BJ, Endicott D, Lewis CC (2018). A method for tracking implementation strategies: an exemplar implementing measurement-based care in community behavioral health clinics. Behav Ther.

[86] Flodgren G, Parmelli E, Doumit G, Gattellari M, O’Brien MA, Grimsshaw J, et al. Local opinion leaders: effects on professional practice and health care outcomes. Cochrane Database Syst Rev. 2011;(8):CD000125. 10.1002/14651858.CD000125.pub4. Published 2011 Aug 10. 10.1002/14651858.CD000125.pub4 PMC4172331 21833939

[87] Proctor EK, Landsverk J, Aarons GA, Chambers DA, Glisson C, Mittman BS (2009). Implementation research in mental health services: an emerging science with conceptual, methodological, and training challenges. Admin Pol Ment Health.

[88] Bhattacharyya O, Reeves S, Zwarenstein M (2009). What is implementation research?: rationale, concepts, and practices. Res Soc Work Pract.

[89] Bauer MS, Damschroder L, Hagedorn H, Smith J, Kilbourne AM (2015). An introduction to implementation science for the non-specialist. BMC Psychol.

[90] Damschroder LJ, Aron DC, Keith RE, Kirsh SR, Alexander JA, Lowery JC (2009). Fostering implementation of health services research findings into practice: a consolidated framework for advancing implementation science. Implement Sci.

[91] Weiner BJ, Lewis CC, Stanick CS, Powell BJ, Dorsey CN, Clary AS (2017). Psychometric assessment of three newly developed implementation outcome measures. Implement Sci.

[92] QSR International Pty Ltd (2010). NVivo qualitative data analysis software; QSR International Pty Ltd. Version 9.

[93] Bernard HR (2011). Research methods in anthropology: qualitative and quantitative approaches.

[94] Forman J, Damschroder L, Jacoby L, Siminoff LA (2008). Qualitative content analysis. Empirical methods for bioethics: a primer.

[95] Padgett DK (2012). Qualitative and mixed methods in public health.

[96] Krippendorff K (2003). Content analysis: an introduction to its methodology.

[97] Palinkas LA, Aarons GA, Horwitz S, Chamberlain P, Hurlburt M, Landsverk J (2011). Mixed methods designs in implementation research. Adm Policy Ment Health Ment Health Serv Res.

[98] Palinkas LA, Horwitz SM, Chamberlain P, Hurlburt MS, Landsverk J (2011). Mixed-methods design in mental health services research: a review. Psychiatr Serv.

[99] Aarons GA, Fettes DL, Sommerfeld DH, Palinkas LA (2012). Mixed methods for implementation research: application to evidence-based practice implementation and staff turnover in community-based organizations providing child welfare services. Child Maltreat.

[100] Saldana L (2014). The stages of implementation completion for evidence-based practice: protocol for a mixed methods study. Implement Sci.

[101] Chamberlain P, Brown CH, Saldana L (2011). Observational measure of implementation progress in community based settings: the stages of implementation completion. Implement Sci.

[102] Craig P, Dieppe P, Macintyre S, Mitchie S, Nazareth I, Petticrew M (2008). Developing and evaluating complex interventions: the new Medical Research Council guidance. BMJ.

[103] Leon AC, Davis LL, Kraemer HC (2011). The role and interpretation of pilot studies in clinical research. J Psychiatr Res.

[104] Potter D, Briggs-King E, Keen L, Amaya-Jackson L, Mannarino A, Cohen J (2015). North Carolina Child Treatment Program Fidelity & Competence Consultation Metric.

[105] Kraemer HC, Mintz J, Noda A, Tinklenberg J, Yesavage JA (2006). Caution regarding the use of pilot studies to guide power calculations for study proposals. Arch Gen Psychiatry.

[106] Moore CG, Carter RE, Nietert PJ, Stewart PW (2011). Recommendations for planning pilot studies in clinical and translational research. Clin Transl Sci.

[107] Thabane L, Ma J, Chu R, Cheng J, Ismaila A, Rios LP (2010). A tutorial on pilot studies: the what, why, and how. BMC Med Res Methodol.

[108] Bowen DJ, Kreuter M, Spring B, Cofta-Woerpel L, Linnan L, Weiner D (2009). How we design feasibility studies. Am J Prev Med.

[109] Bunger AC, Hanson RF, Doogan NJ, Powell BJ, Cao Y, Dunn J (2016). Can learning collaboratives support implementation by rewiring professional networks?. Adm Policy Ment Health Ment Health Serv Res.

[110] Bunger Alicia C., Lengnick-Hall Rebecca (2018). Do learning collaboratives strengthen communication? A comparison of organizational team communication networks over time. Health Care Management Review.

[111] Institute of Medicine (2015). Psychosocial interventions for mental and substance use disorders: a framework for establishing evidence-based standards.

[112] National Academies of Sciences, Engineering, and Medicine (2019). Fostering healthy mental, emotional, and behavioral development in children and youth: a national agenda.

[113] National Institute of Mental Health (2015). National Institute of Mental Health strategic plan for research.

