# Supplementary material for: Improving the implementation and sustainment of evidence-based practices in community mental health organizations: a study protocol for a matched-pair cluster randomized pilot study of the Collaborative Organizational Approach to Selecting and Tailoring Implementation Strategies (COAST-IS)
Source: Implement Sci Commun. 2020 Feb 25;1:9. doi: 10.1186/s43058-020-00009-5 (PMC7207049; doi:10.1186/s43058-020-00009-5)
Supplement: Supplementary file 1 — Additional file 1. Semi-Structured Interview Guide for Aim 1 of COAST-IS Study [file 43058_2020_9_MOESM1_ESM.pdf]

Additional File 1: Semi-Structured Interview Guide for Aim 1 of COAST-IS Study

Thank you for taking the time to meet with me. As you are aware, this study has focused on developing and piloting the Collaborative Organizational Approach to Selecting and Tailoring Implementation Strategies (COAST-IS). COAST-IS is intended to improve implementation and sustainment by supporting organizations in their efforts to address the many barriers to effective implementation that emerge during different phases of the process. However, the primary purpose of this pilot study is to assess how clinicians and organizational leaders in community mental health organizations experience the intervention. Thus, I will ask you a series of questions about your experiences implementing evidence-based practices, the types of challenges and barriers you have faced, and your thoughts and experiences related to COAST-IS. Please be as forthright as possible, as we are fully invested in making COAST-IS as useful as possible, even if it means making substantial changes to the intervention.

**Question**	**Possible Prompts**
TF-CBT Implementation Experience	
-Can you tell me about your experiences implementing TF-CBT over the course of the last year or so?	
-What specific barriers and facilitators have you encountered in this work and how have you addressed them?	
COAST-IS Broad	
-What has been your experience receiving the COAST-IS intervention?	
Principles of Community Engagement	
-To what extent was your work with COAST-IS coaches driven by mutual respect, two-way knowledge exchange, co-leadership/power sharing, and trust	
Acceptability	
-Did COAST-IS meet your personal needs, preferences, or expectations?	-Were there specific aspects or components of COAST-IS that were not aligned with your needs, preferences, or expectations?<br>-Were there specific aspects or components of COAST-IS that were particularly well-matched to your needs, preferences, or expectations?
- What could be changed to make COAST-IS more aligned with your needs, preferences and expectations?	
Appropriateness	
-Did COAST-IS seem to fit within the context of your organization's work and mission?	-In what ways (if any) was COAST-IS a poor fit with your organizational context?<br>-In what ways (if any) did COAST-IS fit well with your organizational context?
-What could be changed to enhance the fit between your organization's needs and COAST-IS?	
Feasibility	
-How easy was it for you to engage in the COAST-IS intervention?	-If it was not easy, what got in the way of engaging fully in the intervention and the associated processes?
-Is there anything that can be done to make COAST-IS more practical for community settings?	
Utility	
-Did COAST-IS increase your ability to identify relevant determinants (i.e., barriers and facilitators) of implementation?	-If so, how?<br>-If not, what were the challenges and what was missing?

-Did COAST-IS increase your ability to identify theory-based change methods and implementation strategies?	-If so, how?<br>-If not, what were the challenges and what was missing?
-Did COAST-IS impact your organization's ability to effectively link strategies to specific barriers?	-If so, how?<br>-If not, what were the challenges and what was missing?
-Was your organization able to develop an overarching implementation strategy that was tailored to address your local concerns?	-If so, what did that look like?<br>-Did that strategy need to change over the past 6 months?<br>-If not, can you tell me more about why no overarching strategy was developed?
-How confident are you that implementation strategies that your organization has used effectively targeted and addressed the most important implementation barriers and facilitators?	-What could have been done to improve that process?
Wrap-Up (Other)	
-If I asked you to redesign COAST-IS (or develop another intervention) to support organizations' efforts to implement and sustain evidence-based practices, what would you include as "core components?"	
-Is there anything else that you would like to share related to your experiences related to implementing trauma-focused interventions, COAST-IS, or anything else that we have discussed today?	
