# Supplementary material for: Improving the implementation and sustainment of evidence-based practices in community mental health organizations: a study protocol for a matched-pair cluster randomized pilot study of the Collaborative Organizational Approach to Selecting and Tailoring Implementation Strategies (COAST-IS)
Source: Implement Sci Commun. 2020 Feb 25;1:9. doi: 10.1186/s43058-020-00009-5 (PMC7207049; doi:10.1186/s43058-020-00009-5)
Supplement: Supplementary file 2 — Additional file 2. COAST-IS Fidelity Tool (Date of Version: 9-19-19) [file 43058_2020_9_MOESM2_ESM.pdf]

Additional File 2: COAST-IS Fidelity Tool (Date of Version: 9-19-19)

	Activity Date	Missing*
**Stage 1: Exploration of COAST-IS**		
Date site is initially invited to participate in COAST-IS [DNS]		
Date COAST-IS introductory materials sent to site [DNS]		
Date site expresses interest in COAST-IS		
Date site confirms understanding expectations for participating in COAST-IS (call or email)		
Date site agrees to participate in COAST-IS (site consents)		
Date baseline barrier assessment (survey) sent to site staff [DNS]		
Date data collection for baseline survey completed		
Date site informed about study condition (COAST-IS vs control) [DNS]		
Did the site discontinue during this stage? If yes, indicate the date the site declined to consider implementation of COAST-IS		
**Stage 2: Preparation for COAST-IS Participation**		
Date roster of site's implementation team provided		
Date site's implementation team members agree to participate in COAST-IS (staff consent)		
Date of introductory Zoom meeting with site's implementation team		
Date site's implementation team agrees to communication plan		
Date site's implementation team agrees to expectations for coaching calls.		
Date site visit schedule finalized		
Date interactive COAST-IS education sessions schedule finalized		
Date coaching calls schedule finalized		
Date of participation in **COAST-IS Session #1** (overview of implementation science and COAST-IS process)		
Date **in-person site visit** conducted		
Date TF-CBT implementation and sustainment goals clarified		
Date of performance objectives brain-storming with site leaders		
Date of performance objectives brain-storming with clinicians		
Did the site discontinue during this stage? If yes, indicate the date the site declined to continue implementation of COAST-IS		

**Stage 3: Implementation of COAST-IS process**		
Date of participation in **COAST-IS Session #2** (review common performance objectives and barriers from across sites)		
Date site-specific report on performance objectives and barriers shared with site's implementation team [DNS]		
Date of **coaching call #1** with site's implementation team		
Date summary of performance objectives generated at site visit discussed		
Date list of performance objectives to focus on finalized		
Date COAST-IS team begins populating Intervention Mapping Matrix [DNS]		
Date of **coaching call #2** with site's implementation team		
Date survey results shared with site and prioritize barriers to address		
Date COAST-IS team continues populating Intervention Mapping Matrix [DNS]		
Date of participation in **COAST-IS Session #3** (overview of implementation strategies to achieve common objectives)		
Date of **coaching call #3** with site's implementation team		
Date feasibility and fit of various implementation strategies discussed		
Date COAST-IS team continues populating Intervention Mapping Matrix [DNS]		
Date COAST-IS team shares populated Intervention Mapping Matrix [DNS]		
Date of participation in **COAST-IS Session #4** (introduce Matrix and tailoring strategies to context)		
Date of **coaching call #4** with site's implementation team		
Date Intervention Mapping Matrix finalized		
Date implementation strategies prioritized		
Date of participation in **COAST-IS Session #5** (overview of implementation plans and how to track and adapt strategies)		
Date COAST-IS team provides implementation plan template (with self-tracking incorporated) [DNS]		
Date of **coaching call #5** with site's implementation team		
Date implementation plan inputs discussed		
Date process for tracking implementation strategies discussed		
Date site's implementation team finalizes **implementation plan**		
Additional coaching calls? [DNS]		
If yes, number of additional coaching calls [DNS]		
Did the site discontinue during this stage? If yes, indicate the date the site declined to continue implementation of COAST-IS		

Stage 4: Sustainment of COAST-IS process		
Date of **coaching call #6** with site's implementation team		
Date active consideration of existing or new barriers indicated		
Date active tracking implementation strategies and monitoring of process toward achieving objectives indicated		
Date active consideration of strategy adaptations indicated		
Date active consideration of new or additional objectives or strategies indicated		
Date of **coaching call #7** with site's implementation team		
Date active consideration of existing or new barriers indicated		
Date active tracking of implementation strategies and monitoring of progress toward achieving objectives indicated		
Date active consideration of strategy adaptations indicated		
Date active consideration of new or additional objectives or strategies indicated		
Date of **coaching call #8** with site's implementation team		
Date active consideration of existing or new barriers indicated		
Date active tracking of implementation strategies and monitoring of progress toward achieving objectives indicated		
Date active consideration of strategy adaptations indicated		
Date active consideration of new or additional objectives or strategies indicated		
Date of **coaching call #9** with site's implementation team		
Date active consideration of existing or new barriers indicated		
Date active tracking of implementation strategies and monitoring of progress toward achieving objectives indicated		
Date active consideration of strategy adaptations indicated		
Date active consideration of new or additional objectives or strategies indicated		
Date of **coaching call #10** with site's implementation team		
Date active consideration of existing or new barriers indicated		
Date active tracking of implementation strategies and monitoring of progress toward achieving objectives indicated		
Date active consideration of strategy adaptations indicated		
Date active consideration of new or additional objectives or strategies indicated		
Date of **coaching call #11** with site's implementation team		
Date active consideration of existing or new barriers indicated		
Date active tracking of implementation strategies and monitoring of progress toward achieving objectives indicated		
Date active conisderation of strategy adaptations indicated		

Date active consideration of new or additional objectivs or strategies indicated		
Date of **coaching call #12** with site's implementation team		
Date active consideration of existing or new barriers indicated		
Date active tracking of implementation strategies and monitoring of progress toward achieving objectives indicated		
Date active consideration of strategy adaptations indicated		
Date active consideration of new or additional objectives or stratgies indicated		
Additional coaching calls? [DNS]		
If yes, number of additional coaching calls [DNS]		
Did the site discontinue during this stage? If yes, indicate the date the site declined to sustain COAST-IS		
